# The Physics of Fluid Dynamics Applied to Vascular Ulcers and Its Impact on Nursing Care

**DOI:** 10.3390/healthcare8020147

**Published:** 2020-05-28

**Authors:** José Miguel Robles-Romero, Macarena Romero-Martín, Gloria Conde-Guillén, Daniel Cruces-Romero, Juan Gómez-Salgado, José Antonio Ponce-Blandón

**Affiliations:** 1 Faculty of Nursing, Department of Nursing, University of Huelva, 21071 Huelva, Spain; josemiguelroblesromero@gmail.com (J.M.R.-R.); macarena.romero@denf.uhu.es (M.R.-M.); 2 Cruz Roja University Nursing School, University of Seville, 41009 Seville, Spain; japonce@cruzroja.es; 3 Ministry of Education, Government of Spain, Delegation of Huelva, 21002 Huelva, Spain; gloria.conde.guillen.11@gmail.com; 4 Industrial Engineering Department, Gabitel Engineers, 21003 Huelva, Spain; daniel.cruces20@gmail.com; 5 Faculty of Labour Sciences, Department of Sociology, Social Work and Public Health, University of Huelva, 21007 Huelva, Spain; 6 Safety and Health Postgraduate Programme, Universidad Espíritu Santo, Guayaquil 092301, Ecuador

**Keywords:** nursing care, microfluidics, physics, venous ulcers, skin ulcers, pressure ulcers, vascular system injuries

## Abstract

The high incidence of vascular ulcers and the difficulties encountered in their healing process require the understanding of their multiple etiologies to develop effective strategies focused on providing different treatment options. This work provides a description of the principles of the physics of fluid dynamics related to vascular ulcers. The morphological characteristics of the cardiovascular system promote blood flow. The contraction force of the left ventricle is enhanced by its ability to reduce its radius of curvature and by increasing the thickness of the ventricular wall (Laplace’s Law). Arterial flow must overcome vascular resistance (Ohm’s equation). The elastic nature of the artery and the ability to reduce its diameter as flow rate progresses facilitate blood conduction at high speed up to arteriolar level, and this can be determined by the second equation of continuity. As it is a viscous fluid, we must discuss laminar flow, calculated by the Reynolds number, which favors proper conduction while aiming at the correct net filtration pressure. Any endothelial harmful process that affects the muscle wall of the vessel increases the flow speed, causing a decrease in capillary hydrostatic pressure, thus reducing the exchange of nutrients at the interstitial level. With regard to the return system, the flow direction is anti-gravity and requires endogenous aid to establish the Starling’s equilibrium. Knowledge on the physics of vascular fluid dynamics makes it easier to understand the processes of formation of these ulcers so as to choosing the optimal healing and prevention techniques for these chronic wounds.

## 1. Introduction

Vascular ulcers are lesions that occur in the lower limbs and they are caused by a problem in the dynamics of distal blood flow, either in the flow of nutrient intake, as in the case of arterial ulcers, or in the return process in the case of venous ulcers.

The high incidence of the onset of these ulcers, 1%–2% of injuries in adults [1], makes it necessary for professionals to adequately manage and know these processes so as to reduce their incidence, to favor the control of risk factors, as well as speeding up healing processes. Venous ulcers affect approximately 1% of the entire Spanish population, becoming 75% of cases of chronic wounds of lower limbs. Its incidence, the difficulties in healing, and the population at risk makes it essential for nursing to know of the etiology of these ulcers, as well as the corresponding appropriate treatment techniques [2].

Vascular ulcers affect the quality of life of people who suffer from them due to their duration, the extent of the wound, and the presence of exudate, odor, and pain. All this decreases the patients’ quality of life, well-being, and capacity to perform their daily life and social activities [3]. Venous ulcers consume a good part of the health budget because of the cost of the products and services associated to them. Although the right treatment is expensive, it is compensated with increasing results in quality of life and acceleration in the healing process [4].

These wounds are caused by an underlying pathology that favors their appearance and hinders their healing. The role of nursing in prevention and healing is crucial, as nurses are responsible for both the prevention and promotion of health for patients at risk, as well as for applying healing techniques and appropriately using materials for the recession of the episode [5]. 

Nursing promotes preventive measures, such as a balanced diet low in sugars and fats that reduces the endothelial harmful effect caused by high concentrations of these nutrients [6] and reducing sodium consumption to prevent the increase of blood pressure caused by its osmotic effect and the consequent affectation of the arterial walls [7]. Nursing also promotes the practice of continued physical exercise that achieves the reduction of body weight, blood pressure by adaptation of the smooth musculature, and the increase of microvascular regeneration [8].

The pathophysiology of venous ulcers differs from that of arterial ulcers. Therefore, preventive and healing measures vary depending on their etiology. Compression therapy is a first-choice treatment for venous ulcers. It consists of applying distal-to-proximal descending pressure with a bandage, from the foot to the patellar area [9]. However, in the case of arterial ulcers, this kind of bandage is contraindicated in all cases due to the associated increase in the ischemic effect [10]. Adhesive drapes are contraindicated for venous ulcers because of the weakness of the perilesional skin and excessive interstitial pressure. In the case of arterial ulcers, these drapes can be used without any caution, since they are characterized by in-depth advance, and not in extension, so the surrounding tissue is not compromised [6]. Most venous ulcers are very painful, unlike arterial ulcers, limiting the use of surgical debridement to very rare cases [5].

The diversity of the treatment depending on the ulcers’ etiology makes it necessary to know their pathophysiological process and the physical laws that condition them. This knowledge will allow to choose between the treatment options, both preventive and healing, for a more effective approach [11]. A relationship between the laws of physics and pathophysiological processes together with their consequent nursing care has already been showed. The effect of Newton’s third law has been identified in the immobilization of a child’s arm with a channeled peripheral pathway [12]; the postulates on matter and energy have served as the basis for explaining the field of human energy [13], and the principles of quantum physics have been used as a theoretical framework for communication and interpersonal relationships in healthcare settings [14]. However, physical foundations related to vascular ulcers have not been described.

This work aims to describe the principles of the physics of fluid dynamics related to vascular ulcers to help understand their pathophysiology and choose the best treatment option. All the equations displayed in this article are compiled in the Supplementary File 1.

## 2. Methods

A review of literature was conducted. An electronic search was carried out in healthcare databases such as Medline, Web of Science, and Scopus, combining the key words “physics”, “fluid dynamics”, “skin ulcer”, “vascular ulcer”, “physiology”, and “nursing care”. Articles published in the last ten years, written in English or Spanish, were selected. They were critically analyzed, and data was extracted in order to meet the aim of the study.

## 3. Discussion

### 3.1. Cardiovascular Physiology

The human body presents an anatomical design that is used by the principles of terrestrial physics so that bodily processes can occur in the most effective and efficient way possible. The tissue irrigation system is favored in its function by anatomical intrinsic factors, and it takes advantage of its design and of the principles of physics and the postulates of fluid dynamics. The human cardiovascular system consists of a circuit of arteries and veins of more than 100,000 km in length, which are travelled by a blood volume of approximately 5 liters, divided standing into 84% in major circulation (of which 74% is placed in the venous system), 9% in minor circulation, and 7% in the heart [15].

The process of nutrient distribution is performed by blood vessels, which show anatomical differences depending on their characteristics and purpose. In general, we can distinguish between arteries, veins, and capillaries, which are responsible for the tissue exchange. Arteries and veins are made up of three layers or tunicae [16]:

- External or adventitia. Made up of collagen, which coats the vessels, thus providing structural support and shaping.
- Media. Composed of muscle tissue with high content of elastin and collagen fibers, surrounded by elastic tissue. The proportion of these tissues varies depending on the diameter and function of the vein or artery. This layer, in the case of the arterial tree, is responsible for bearing the necessary pressures to send blood to all tissues. It has a vasoconstrictor capacity that helps increasing interstitial products exchange, as well as blood redistribution according to body needs.
- Inner layer or intima. Formed by an endothelium that coats the vein or artery and that is in contact with the blood fluid. It is responsible for reducing friction at the passage of the blood fluid.

### 3.2. Ventricular Morphology

The blood is driven by the heart pump, which causes its movement to achieve the necessary speed for the interstitial exchange to occur. Although both ventricles exert pressure, they show differences. The left ventricle is subjected to high peripheral resistance, so it has a greater ability to cause high blood pressure. The greater thickness of its free wall, coupled with the decrease of the radius of curvature during the contraction, increases the reduction of blood pressure during contraction, causing a greater blood pumping force [17] and presenting a less prolonged ejection fraction that leads to an isovolumetric relaxation phase 55% higher than that of the right ventricle.

**Figure 1:**
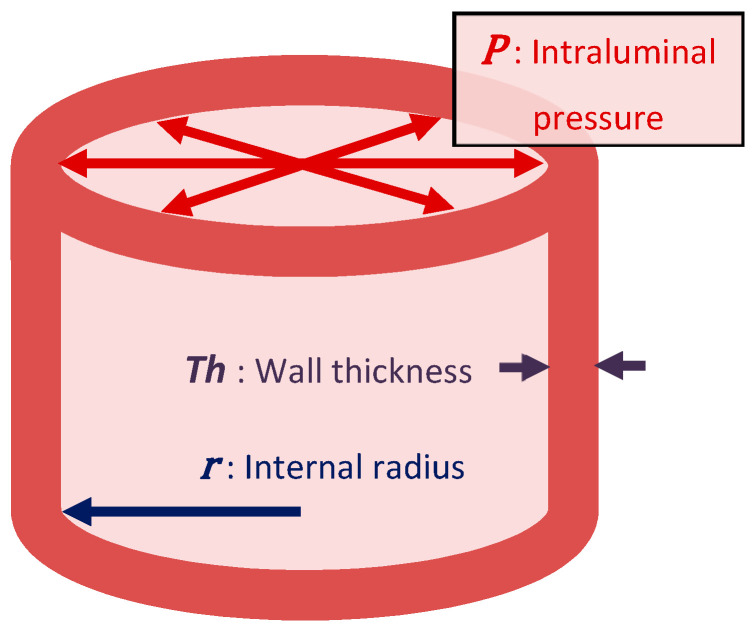
Laplace’s law.

The Laplace’s Law (see Figure 1) explains the relationship between the pressure of the arteries and their diameter. In cardiac terms, when the systole occurs, the radius of curvature is reduced, so the internal pressure, which is always higher than the external one, increases as this diameter decreases. Thus, the blood is more strongly driven to the large vessels [18]. Therefore, if we take into account ventricular morphology and know that wall tension (*T_w_*) is the force that blood will exert to try to separate actin and myosin myofibers, this pressure will depend on the internal radius (*r*) of the round surface of the ventricle or vessel, on transmural pressure (*P_t_*) and, in an inversely proportional way, on the wall thickness (*Th*). Hence, ventricular geometry can be a good predictor of cardiac functionality, since any pathology that causes ischemic or hypertrophic injury and that involves a change in its shape directly affects the dynamics of fluids causing, at the capillary level, a deficit of nutrient intake or an accumulation of interstitial fluid, with the consequent risk of distal injury [17].

$$T=\frac{P\times r}{2Th}$$

*T*: Wall tension; *P*: Intraluminal pressure; *r*: Internal radius; *Th*: Wall thickness

$$T_{w}=\left(P_{t}\cdot r\right)/2Th$$

*T_w_* = wall tension; *P_t_* = transmural pressure; *r* = radius of the ventricle; *Th* = wall thickness

In the case of pathology that causes coronary hypertrophy, the size of the ventricle increases and, therefore, *r*:

Δr≫0→ΔT≫0⇒Harmful coronary effect

In the case of continuous physical exercise practice [19], the ventricular wall thickness (*Th*) increases:

$$\Delta Th\gg 0\to {\;}^{\infty }\Delta T\ll 0\Rightarrow Protective\;coronary\;effect$$

### 3.3. Arterial Morphology

The arterial wall is thicker than the venous wall and its cross section is smaller than that of its corresponding vein, as arteries are subjected to high pulse pressure. To be able to support this pressure, they have a high percentage of elastic tissue and less smooth musculature. The high concentration of elastin gives arteries the option to modify their diameter as needed. As they approach the organs or tissues for irrigation, they decrease in size to become arterioles (8–60 micrometers), lowering the ratio of smooth musculature to increase muscle ratio. These have vasoconstrictive or vasodilating abilities thanks to the autonomic nervous system, thus being able to meet the oxygen demands of tissues [19]. They are vital as they cause peripheral vascular resistance, which is essential when understanding chronic ulcerative pathophysiology.

Therefore, we find a viscous fluid conduction system (blood has a viscosity of 3.5 > water) [20] that decreases its diameter as it moves away from the heart, withstanding high pressures and decreasing flow rate. The term flow rate (*F*) influences tissue irrigation capacity and is defined as the circulating blood volume through a cross section of a vessel in a time unit (volume/minute).

$$S=\frac{F}{\pi }\cdot r^{2}$$

*S =* blood speed; *F =* flow rate; *r =* radius of the vessel

Another important aspect to consider is the damping effect of the pulse flow sent by the left ventricle (*LV*). The blood reaches the vessels intermittently and, as it descends in level and reaches the arterioles, the flow becomes continuous and slow at the capillary level, from 400 mm/s in aorta to 0.1 mm/s in capillaries. This effect is essential for the tissue irrigation process, and to a greater extent in distal areas, because if the speed at which the blood circulates is faster than normal, the hydrostatic pressure (*P_h_*) would decrease until disappearing, implying no exchange, with the consequent ischemia of irrigated tissues by affected arterioles and its characteristic injury [21].

$$P_{h}=d\times g\times h$$

*d* = blood density; *g* = gravity; *h* = height

In the case of arterial ulcers, the capillaries lose their elasticity, which directly affects blood pressure (*P_b_*), understood as the force exerted by the passage of blood against the vascular walls per unit surface, measured in mmHg. This pressure is directly proportional to cardiac output (*CO*), and inversely proportional to the elasticity of the arterial walls (*e*), that determine peripheral resistance (*Rp*) [21].

Δe≪0 → ΔRp≫0 → ΔBp ≫0

Therefore, in arterial ulcers, the presence of distal pulse is a sign of possible recovery. Otherwise, it would indicate arteriolar stiffness with the consequent alteration of *P_h_* and the ineffective exchange of nutrients at interstitial level.

### 3.4. Venous Morphology

The veins also have the three layers mentioned above, but unlike the arteries, the pressure to be endured is much lower, so their walls are much thinner and with a lower proportion of muscle and elastic tissue. However, the cross-sectional diameter is elliptical and larger than its corresponding artery [21]. This return system has a high capacity to withstand large volumes with very low pressure, so the fluid dynamics is completely different from that of the arteries. This circuit is responsible for collecting blood from the capillaries, which increases the diameter of the ducts. Hence, it does not rely on the same principles of physics as the arterial tree.

Regarding gravity, in the venous system, the blood rises in an anti-gravity direction. Although gravity affects the whole system, it is in the distal areas where it has the greatest effect, increasing capillary *P_h_* and favoring the cluster of interstitial fluid. The venules, of less thickness and lower resistance and more likely to be broken by excess volume, collect the capillary blood and lead it to the larger veins (approximately 70% of the blood volume) at a much lower speed than the arterial system does, until reaching the vena cava, of about 3 cm diameter. The venous system has a much slower circulation speed than the arterial one, reaching about 10–15 cm/s at rest until approximately 50 cm/s during physical exercise [22].

The veins have anti-return valves, approximately every 2–4 cm, formed by retreats of the intimate tunic itself, that is oriented to the heart to favor the return process, and is also helped by muscle pumps. The venous flow comes from the capillary bed; it is continuous and rises to middle areas where small oscillations may appear, but the characteristic arterial pulse always differs.

### 3.5. Capillary Morphology

In capillaries, the physical processes that cause product exchange between the vascular-interstitial spaces occur. This is because they only present an endothelial layer surrounded by a basal membrane that makes it permeable to the passage of fluids and metabolites, but not of large molecules. They usually measure 1 mm and have a diameter of approximately 5–10 microns, in many cases smaller than erythrocytes (8 microns). In an average body, the tissue exchange surface is around 700 m^2^, with no more than 20 microns distance between capillaries and cells [21].

### 3.6. Blood Haemodynamics

In addition to vascular morphology, fluid hemodynamics is another determining factor in the pathophysiology of vascular lesions.

### 3.7. Arterial Flow Dynamics

**Figure 2:**
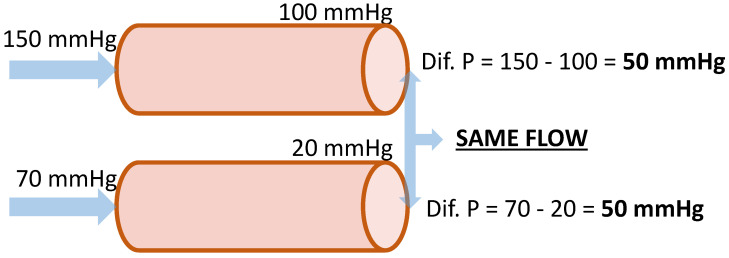
Gradient of a fluid pressure.

Blood flow begins in the aorta, the main artery with a diameter of approximately 2.5 cm. Blood, like any fluid, moves through the arteries driven by pressure differences, moving from the highest pressure zone (P_1_) to the lowest one (P_2_), depending on the gradient between both areas, i.e., the difference between the starting area and the final area (see Figure 2). The area that withstands the greatest pressure is the closest one to the heart, the aorta artery, and is the one with lowest number of capillaries. This dynamic causes blood movement and facilitates nutritional exchange in the capillary areas. In blood flow, vascular resistance (*R*) is worth considering, always moving in the opposite direction to movement. Its increase influences the development of ulcerative lesions in lower limbs, as it affects arterial flow rate (blood volume per time unit) [16]. Mathematically, it is defined through the Ohm’s equation:

$$F=\frac{\Delta P\left(P_{1}-P_{2}\right)}{R}$$

*F* = arterial flow rate (volume per time unit); *P* = pressure; *R* = vascular resistance

Therefore, if resistance increases, the flow decreases, resulting in a higher risk of vascular ulcers:

If ΔR ≫0→ ⇙F   If ΔP ≫0→ ⇗F

Vascular resistance allows to assess the friction caused by molecules moving against the vessels’ wall. This, in turn, depends on two variables: the diameter of the artery and the characteristics of the fluid. As for the arterial diameter, when the blood circulates inside an artery, its speed (*s*) is greater as the flow increases (*ΔF* ≫0*→ Δs* ≫0), and lower as the radius of the artery increases (*Δ*r ≫0*→ Δs* ≪0). Mathematically, it can be understood and calculated by applying the second continuity equation:

$$A_{1}\cdot S_{1}=A_{2}\cdot S_{2}$$

*A =* area of the duct; *S* = blood speed

Therefore, we can determine that *F* depends on the area of arterial lumen and on the flow speed, so by decreasing these areas, the blood speed should increase:

If ΔA ≪0→ΔS≫0

In relation to the characteristics of the fluid, viscosity must be taken into account (*Π*). This is understood as the property of exhibiting resistance to tangential displacement of molecules’ layers and the type of flow. Blood viscosity should be considered as a non-Newtonian fluid as it is composed of plasma and cells of different sizes. Cellular makeup is measured through hematocrit, which indicates the percentage of erythrocytes in blood volume [16]. Resistance to movement will increase with the increase in the number of cells. That is, having a high hematocrit (normal: 40.7%–50.3% for men and 36.1%–44.3% for women) and higher density (normal density: 1060–1088 kg/m^3^) may be a vascular risk factor due to the increased thrombotic effect. Viscosity is conditioned by speed, as this will decrease *Π* due to the higher concentration of large molecules in the middle. Hence, ⇑ hematocrit → ⇑ density → ⇓ speed → ⇑ thrombotic effect [23].

Under normal conditions, as blood speed increases, cells are reordered from largest to smallest in size from the center outwards, with the blood cells placed inside the current and plasma on the outside. This model is known as laminar flow. In case of excess speed, swirls occur inside the vessel, causing the pressure to decrease in the wall and favoring the coagulation cascade activation. This type of movement is called turbulent flow, being a risk factor to consider for the appearance of thrombus [23].

The flow type is calculated using the Reynolds number (*N_r_*), knowing that laminar flow is considered if *N_r_* < 2000, and turbulent flow if *N_r_* > 2000. The following is formulated:

$$N_{r}=\frac{2r\times S_{m}\times d}{\Pi }$$

*r* = arterial radius; *S_m_* = mean speed; *d* = density; *Π* = viscosity

Another value to consider is the shear stress supported (*τ*) by the arterial endothelium at the passage of blood. Its normal values range from 0.009 mmHg to 0.019 mmHg and, in cases of lower pressure, the appearance of atheromatous plaques with the consequent cardiovascular risk is favored. This risk arises from vessel occlusion or from the increase of *Ss* in the proximal area of the plaque, due to the produced stenosis and the increased risk of vessel rupture, caused by the reduction of the fibrous layer.

$$\Pi =\frac{\tau }{\Delta S}$$

*τ* = shear stress or propelling tension; ΔS = speed gradient between fluid layers

**Figure 3:**
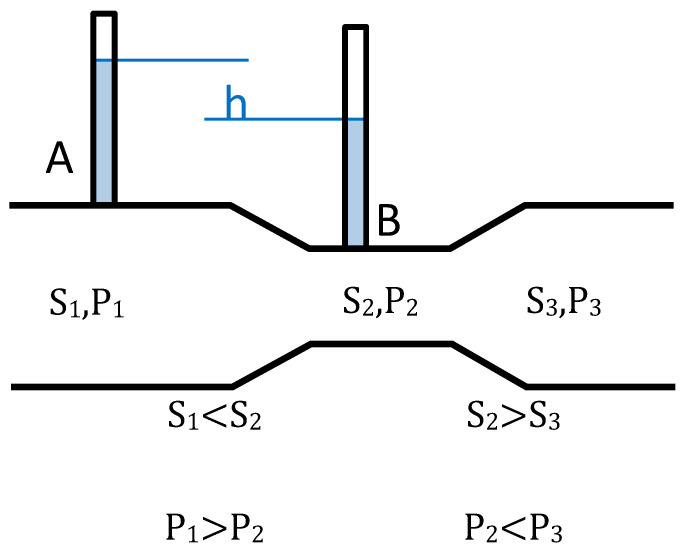
Representation of the Venturi effect. S: speed; *P*: pressure.

Blood is sent by impulses through the ventricle, and arteries are responsible for converting intermittent flow (ventricle–aorta) into continuous flow (arterioles–capillary circulation). This is thanks to the Windkessel effect, which transforms the potential energy produced by the deformation of the aortic wall into kinetic energy to push blood through arterioles, capillaries, and venules continuously. Another principle to consider in relation to vascular ulcers is the Venturi effect (see Figure 3), which states that when a fluid is circulating through a duct and its radius (*r*) is reduced, the speed (*s*) of the fluid increases and the pressure (*P*) decreases.

### 3.8. Venturi Effect

If⇙ r→ Δs ≫ 0→ΔP ≪ 0

This effect is mainly applicable in the case of stiffening at distal arterial level. Thus, the throbbing flow will disappear. The distal artery becomes a rigid tube that reduces in size as it progresses, increasing blood speed in capillaries and reducing *P_h_*, with the consequent tissue ischemia occurring in arterial ulcers. This effect is more pronounced in the presence of atheromatous plaque, since the reduction of the arteriolar diameter favors the increase of blood speed and the reduction of pressure to such an extent that it can cause occlusion due to the suction effect. The Bernoulli equation considers the relationship between the effects of pressure, speed, and gravity, being key in coronary ischemic processes due to the passage of blood through a narrowed area [23].

$$\frac{S_{1}^{2}}{2g}+\frac{P_{1}}{y}+Z_{1}=\frac{S_{2}^{2}}{2g}+\frac{P_{2}}{y}+Z_{2}$$

*g* = gravity; *P* = pressure; *y* = specific weight (constant in incompressible fluids); *Z* = height level

### 3.9. Capillary Flow Dynamics

The capillary area is where the exchange between the intravascular space and the cells occurs, using the interstitial zone as a connection. The blood speed is slower so that the processes can occur, varying from about 40 cm/s in the aorta, to 0.01 cm/s. The speed is inversely proportional to the *P_h_* caused against the vessel wall. Therefore, the slow and continuous capillary blood speed leads to the appearance of higher pressure [23]. That is, any factor that increases blood speed in the capillary will imply a lower supply of nutrients to the area and the consequent tissue injury.

Blood tissue is in motion, and the exchange occurs in both vascular and interstitial directions thanks to the porous structure of the vessel wall, which is formed by a small layer of endothelial cells and a small lining basal membrane. This, together with the small diameter of the pore, favors the plasma that is rich in nutrients and oxygen to go outside (filtration) in the arterial capillary, and, in turn, waste products to re-enter the venous system (reabsorption) in the venous capillary. This exchange depends on two types of opposite gradients: one exercised by circulating fluid (hydrostatic), and one by proteins (colloid osmotic). These proteins are found in higher concentration within the vessel, as capillary permeability does not allow outflow. Around 70% is caused by albumin which is synthesized by the liver and accounts for more than 50% of plasma proteins. The remaining 30% is caused by globulins and fibrinogen, so in case of acute patients with protein deficit, a fluid outflow to the interstitium and the consequent edema are produced, which may even lead to anasarca. Both gradients are bidirectional (interstitial⇔vascular) [24]. These forces must remain in constant balance for a correct exchange process. This is known as Starling’s equilibrium and must occur according to four factors [16]:

- Capillary hydrostatic pressure (*P_ch_*). It is exerted by plasma fluid in contact with the endothelium. Its value ranges from 35 mmHg at the arterial end to 15 mmHg in the venous end.
- Interstitial colloid pressure (*P_IC_*). It is exerted by interstitial fluid on the vessel, and it is usually close to 0 mmHg; it may be negative due to the lymphatic system. If this magnitude is greater than the internal one, a collapse of the capillary is caused, with the consequent lack of nutrients in the area, as could occur in the case of severe distal edema or by compression that prevents normal return.
- Colloid, oncotic, or capillary osmotic pressure (*P_CC_*). The osmotic effect of proteins inside the vessel causes fluid to enter the inside, usually around 28 mmHg, and it is mainly produced by albumin.
- Interstitial colloid or osmotic pressure (*P_Ic_*). Caused by proteins found in the interstitium, in smaller quantities due to the low capillary permeability to large molecules under normal conditions, with an approximate value of 3 mmHg.

The sum of all pressures that occur in the capillary causes the movement of intraspace fluids, where the resulting pressure, or effective/net filtration pressure (P_F_) is:

$$P_{F}=\left(P_{CH}+P_{IC}\right)-\left(P_{IH}+P_{CC}\right)$$

*P_CH_* = capillary hydrostatic pressure, *P_IC_* = interstitial colloid pressure, *P_IH_* = interstitial hydrostatic pressure, *P_CC_* = capillary colloid pressure.

Therefore, under normal conditions, we can distinguish a flow in the arterial capillary area, where the fluid that is pushed out is greater in volume than the one pushing inwards, resulting in filtration; and a flow in the venous area, where the external pressure is higher than the internal, causing the fluid to enter the capillary, a process called reabsorption.

If we consider the fact that filtration has a higher potency than reabsorption, one might think that excess fluid would always exist. Therefore, in this complex balance of fluid movement, the lymphatic system is responsible for collecting the excess that is not reabsorbed and returning it to the venous system [16]. Daily, a filtration of approximately 20 liters is produced, of which 18 liters are absorbed through the venous capillary and 2 liters through the lymphatic system. It is therefore important to consider the lymphatic system when choosing the healing treatment for chronic ulcers, taking into account its effect on pressure processes, raising of limbs, and materials to be used.

### 3.10. Venous Flow Dynamics

Once the blood is collected from the capillaries with all the waste products, it enters the venous system from the venules with lower caliber to converge in larger vessels, until reaching the vena cava, which will introduce the blood into the right atrium. Unlike arterial fluid dynamics, which can increase or decrease flow as appropriate, venous flow rate dynamics require mechanisms to help return blood volume effectively and efficiently. The distribution of the venous tree ranges from small venules to large vessels, and the flow is antigravity. Therefore, it requires mechanisms that favor this effect: venous valves and body pumps [2].

Venous valves are formed by retreats of the intimate tunic oriented towards the heart and separated approximately every 2–4 cm, in such a way that they prevent the blood from receding and also Δ*P_h_* ≫ 0 in lower sections.

Muscle pump refers to the anatomical design of the body by which it performs distal-to-proximal muscle contractions in all cases, thus favoring the closure of the lower valves and the opening of the upper ones, in turn pushing the blood upwards.

The respiratory pump is activated with inspiration. The negative pressure that occurs in the intrathoracic cavity absorbs the blood of the jugulars, and at the same time the descent of the diaphragm causes the increase of intra-abdominal pressure, favoring the return of blood from the lower limbs to the chest.

The heart pump, referring to the negative pressure that occurs in the right atrium when emptying, causes a suction effect at the level of the vena cava.

In relation to the speed at which blood circulates, although in the arterial zone it is throbbing in the large arteries and continuous at the arteriolar and capillary level, the same thing happens in the venous system, but at a much lower speed and pressure. As for the gravitational effect on the blood circuit, while the arteries of lower limbs are aided by this effect thanks to the downward direction of the flow, in the case of the venous system, gravity works against it, making hydrostatic pressure appear due to the weight of the blood itself, causing blood to move downwards. This provokes that, in venous distal and dependent areas, these are much more prone to fluid cumulus in the interstitium, given that favoring mechanisms are not used properly. Increased peripheral venous pressure implies a decrease in the reabsorbed volume in the capillary beds, which could reduce blood volume by up to 5%. This decrease, however, is counteracted by increased pressure of interstitial fluid, which reduces filtration from the capillary and increases lymphatic drainage [20].

### 3.11. Lymphatic Flow Dynamics

The lymphatic system plays a crucial role in body water balance. It is characterized by an open circuit, formed by a network of small-caliber capillaries that begin in the form of an open sac, and through which both fluids and large molecules can enter. The cell distribution of its endothelium, in the form of a gate, allows for the opening into the capillary to widen as the interstitial fluid pressure becomes higher, this way facilitating a greater passage of lymph (name of the interstitial fluid as it enters the lymphatic circuit) to the torrent. As the lymph rises, the vessels grow in size and thickness, heading towards the chest and right lymphatic ducts and interspersing many nodes at its passage, which work as defensive filters. The large lymphatic vessels flow between the intersections of jugulars and subclavian veins, pouring the lymph back into the venous system [1]. This water recovery system uses similar mechanisms to the venous system to overcome the force of gravity and move upwards through levels until reaching the end of the duct at the subclavian level. The muscle and respiratory pump are undoubtedly the main ones, together with arterial pulses, that also help this antigravity return [23].

### 3.12. Physiopathology of Vascular Ulcers

The arrival of nutrients and oxygen to cells is done through the physical and anatomical processes mentioned above. We start from a closed vascular circuit that is in charge of carrying and collecting components to keep all body tissues alive. Therefore, there are two possible causes of injuries: those that are caused by insufficient arterial irrigation (arterial ulcers), and those that are caused by a deficit in the venous return (venous ulcers) [6].

### 3.13. Physiopathology of Arterial Ulcers

It should be kept in mind that high serum concentrations of solutes are harmful for the vascular endothelial layer, the more important being the more episodes occur of the vascular layer. In this type of lesions, the most common example is that caused by high blood glucose figures over time, as is the case in diabetic patients (more quickly in type I). This hyperglycemia, continued and with recurrent peaks, produces the appearance of corrosive effects that favor inflammatory processes within the vessel lumen and that trigger the effect of altering normal arterial flow in the distal areas, with the consequent risk of injury and/or making recovery difficult.

The physiological process begins with the involvement of the endothelial wall in the first instance, producing an inflammatory effect and its thickening. The progression of the injury ends up damaging the elastic tissues of the arterioles in the medium–long term, thus causing an ineffective regulation of pressure. This process makes blood circulate in the capillaries at a higher speed than necessary, resulting in lower *P_h_* than required. Therefore, there is no plasma output into the interstitial space; that is, arterial ulcers are caused by a nutrient deficit in the injured area, with arteriosclerosis being the main cause of the pathological process [25], reaching 8% of wounds in limbs [7]. Diabetes, smoking, hypertension, and dyslipidemias are the main pathologies that cause endothelial injury in the distal arteries, because of the endothelial corrosive effect and the favoring of the inflammatory effect. Affectation can progress to more important vessels, even inducing nutrient deficit to result in irreversible tissue ischemia at increasingly proximal levels.

With regard to symptomatology, these lesions are characterized by [6]:

- Small, deep in size, with rounded and defined edges and dry bottom, since the lack of arteriolar irrigation induces the death of the cells that required to be nourished by the said vessel.
- High difficulty in regenerating granulation tissue due to the lack of interstitial exchange caused by a decrease in *P_h_* due to an increase in the capillary blood speed.
- Location in pre-tibia areas, metatarsal areas, and fingertips.
- Pale and hairless surrounding skin as a result of a lack of nutrients in all surrounding tissues.
- Absence of arterial pulses due to the stiffening of the arteries’ muscle wall.
- Painless injury bed due to the peripheral neuropathies typical of this pathology.

### 3.14. Physiopathology of Venous Ulcers

These types of lesions affect the venous system and are caused by the weakening of non-return valves (venous insufficiency), with risk factors related all of them to unhealthy lifestyle habits (hypertension, smoking, obesity, sedentary lifestyle, etc.). It should be considered that venous circulation lacks the help of ventricular pressure or gravitational effect. Therefore, it requires efficient body pumps that facilitate the process, mainly muscle pumps in the case of the most distal areas. Therefore, if the patient leads a sedentary life and is overweight, which increases abdominal pressure and prevents normal blood flow, blood speed slows down, and capillary *P_h_* increases in lower limbs’ vessels. As a result, there is a decrease in capillary reabsorption (*P_IH_ + P_CC_*) that occurs in the venous portion and an increase in the amount of fluid in the interstitium, causing edema of the affected leg. This, coupled with the muscle pumping deficit that occurs due to the lack of physical activity these patients usually present, causes the skin to appear thin and fragile, which facilitates the occurrence of a wound. Edema tends to accumulate in the dependent areas of lower limbs (compromising in a greater or lesser degree those cells that are close to the skin) due to the difficulty that nutrients have to reach these areas that are located further away from the capillaries [8]. As a result of this process, the lesions always advance in extension, but, unlike arterial ulcers, not in depth, being also very painful because of the innervation of the first layers of the skin.

Based on the above stated, the main characteristics of this type of injuries are [26]:

- Irregular edges and no definition.
- Flat wounds that grow in extension, but not in depth.
- Normally located between the malleolar area and the knee, starting preferably in lower areas.
- Very painful to palpation and/or manipulation.
- Perilesional edematized, cyanotic and very fragile skin, with poor irrigation.
- May present granulation tissue in the healing process, unlike arterial ulcers.

## 4. Conclusions

The basic principles of the physics of fluid dynamics facilitate the functioning of the circulatory system. The arterial system is aided by the high percentage of elastic tissue of the arteries to transform the rapid pulse flow at the aortic level into continuous flow at the capillary level. There, the speed must be slow so that hydrostatic pressure is higher, and the filtration process is effective. The venous system is hampered by the force of gravity, requiring anti-return valves and body pumps to effectively carry out their function. Arterial ulcers are caused by an injury in the muscle wall of the arterioles that imply a reduction in capillary filtration. For this reason, these are characterized by being small and deep in growth, as they would cause ischemia of a certain area. Venous ulcers are caused by venous insufficiency that leads to a fluid build-up in the interstitial space (edema), reducing reabsorption. 

The nursing field must be acquainted with the physics of fluid dynamics to better understand the physiological process of vascular ulcers. Such knowledge would contribute to more efficiently assimilating the effective procedures and techniques for the healing process, the materials to be used in the clinical practice according to the characteristics of both the patient and the injury, as well as the prevention of possible relapses [8].

In order to obtain therapeutic success, it is also crucial to carry out a thorough record of the procedures performed, facilitating the monitoring and continuity of care, as well as effective and efficient communication with the patient. Fluid communication with the patient where the nurse conveys the principles that explain the etiology of the ulcer and the fundamentals of the clinical practice will help patients better understand their health issues and cope with the slow recovery process. It is essential for the patient to be aware of the risk factors that can lead to the onset of a vascular ulcer, as well as of the recurrence of any already healed ones. These steps ensure higher success rates in our care practice.

This study presents certain limitations related to the methodology of the literature review. In line with the objective of the work, and given the rigor of the search, content and significance of the information were prioritized. 

## Supplementary Materials

The following are available online at https://www.mdpi.com/2227-9032/8/2/147/s1.
