# Supplementary material for: The Physics of Fluid Dynamics Applied to Vascular Ulcers and Its Impact on Nursing Care
Source: Healthcare (Basel). 2020 May 28;8(2):147. doi: 10.3390/healthcare8020147 (PMC7349071; doi:10.3390/healthcare8020147)
Supplement: Supplementary file 1 [file healthcare-08-00147-s001.pdf]

healthcare

MDPI

List of equations

Equation	Symbols	Physiologic application	Nursing application
$T_w = (P_t \cdot r)/2Th$	T_w = wall pressure<br>P_t = transmural pressure<br>*r* = ventricle radius<br>*Th* = wall thickness	Wall pressure measurement (P_w) according to its radius *(r)* and its ventricular wall thickness *(Th)*	Importance of continued physical activity as a preventive measure due to an increase in *Th* and no pathological ventricle hypertrophy *(r)*.
$S = \frac{F}{\pi} \cdot r^2$	*S* = blood speed<br>*F* = flow rate<br>*r* = vessel radius	Blood speed measurement *(S)* according to flow rate *(F)* and artery radius *(r)*	Knowledge on how vasodilation and vasoconstriction effects have a direct impact on tissue irrigation. Controlling temperature of lower limbs to manage vascular effects.
$P_h = d * g * h$	P_h = hydrostatic pressure<br>*d* = blood density<br>*g* = gravity<br>*h* = height	Knowledge on hydrostatic pressure (P_h), which facilitates or impairs capillaries-intesrtitium-capillaries nutrients exchange.	Importance of elevating lower limbs and using compressive measures to reduce oedema in vascular venous pathologies. Also, the importance of contraindications in arterial pathologies due to the increase in capillary blood speed and tissue ischemia.
$F = \frac{\Delta P(P_1 - P_2)}{R}$	*F* = arterial flow<br>*P* = pressure<br>*R* = vascular resistance	Determining blood flow *(F)* according to vascular resistances *(R)* and difference in an artery pressure (ΔP)	Recommendations on healthy life habits to reduce ΔR due to loss of arterial elasticity and subsequent decrease of blood flow.

$A_1 * S_1 = A_2 * S_2$	A = duct area<br>S= blood speed	Blood flow rate depends on the area of arterial lumen *(A)* and of its speed *(S)*	Contraindication of compressive measures as treatment for arterial ulcers due to ΔR and modification of blood speed.
$\Pi = \frac{\tau}{\Delta S}$	τ = shear stress or propelling tension<br>S = speed gradient between fluid layers	Blood viscosity (Π) depends on shear stress (τ) in vascular walls and on the increase in blood speed *(S)*	Importance of favouring adequate blood return measures to avoid a decrease in S and the subsequen t $\Delta\Pi$ and increase of thrombotic effect. Also, knowledge on the importance of the increase in haematocrit as a risk factor due to a slowdown in blood flow.
$N_r = \frac{2r * S_m * d}{\Pi}$	N_r = Reynolds number<br>r = radius<br>S_m = mean speed<br>d = density<br>Π = viscosity	Determination of the type of blood flow, dependent on the vessel radius *(r)*, blood speed *(S)*, density *(d)* and viscosity (Π)	Favouring laminar flow reduces the thrombotic effect, so arterial speed must be promoted without compressive measures that affect r and/or reduce S, as well as appropriate hydration that decrease d.
$\frac{S_1^2}{2g} + \frac{P_1}{y} + Z_1 = \frac{S_2^2}{2g} + \frac{P_2}{y} + Z_2$	g = gravity<br>P = pressure<br>y = specific weight (constant in incompressible fluids)<br>Z = height level	Relationship between pressure effects *(P)* that provoke blood speed *(S)* and gravity *(g)*	Importance of cardiovascular preventive measures in case of arterial endothelial lesion, since it produces stiffness and blood ΔS with a decrease in P_h and ischaemia. Also, in case of a decrease in vessel lumen (by atheromatous plaque, clot, etc.), there is a possible collapse by areal blood ΔS and a decrease in pressure. This also applies to the use of syringes for cleaning and

			clearance of vessels, or for cleaning through drag-out.
$P_F = (P_{Ch} + P_{IC}) - (P_{IH} + P_{CC})$	P_{CH} = capillary hydrostatic pressure<br>P_{IC} = interstitial colloid pressure<br>P_{IH} = interstitial hydrostatic pressure<br>P_{CC} = capillary colloid pressure	Knowledge on the movement of intraspace fluids in the capillary, allowing to determine the effective filtration pressure (P_F), determining the existence or not of oedema.	Determination of the actual cause of the oedema, through variation in P_h, giving proof of endothelial lesion and/or intra o extravascular protein alterations. Also considered in case of lymphadenectomy due to impairment of the lymphatic system.
